# Editorial: Exploring the dynamics of medium and small scale sport events: implications for communities and development

**DOI:** 10.3389/fspor.2026.1900077

**Published:** 2026-07-08

**Authors:** Lavinia Falese

**Affiliations:** 1 University of Cassino and Southern Lazio, Cassino, Italy; 2 European University of Technology EUt+, European Union

**Keywords:** community engagement, economic impact, event sustainability, events impact, small and medium scale sport events, social impact, sport tourism

Medium and small scale sporting events have increasingly attracted attention as vehicles for community development, as they offer opportunities that differ from those associated with mega-events. While large international competitions often dominate public and academic discussions, smaller events are typically more embedded in the everyday realities of host communities. Their success depends on close interactions among residents, athletes, spectators, volunteers, schools, sports clubs, sponsors, tourism stakeholders, and public authorities.

The contributions to this Research Topic show how medium and small scale events should not be viewed simply as scaled-down versions of mega events. Rather, they operate as complex, place-based systems capable of generating a wide range of economic, social, cultural, environmental, technological, and health-related outcomes.

One of the central themes running through the collection is the relationship between event experiences and local value creation. Rossini et al. examine how a medium-sized university sporting event can generate tangible economic benefits for the host community. Their findings suggest that economic impact is shaped not only by attendance numbers but also by factors such as visitor satisfaction, transport accessibility, interactions with event staff, consumption of local products and services, and the duration of participants' stays. The study provides a useful reminder that meaningful economic contributions often emerge through the quality of the overall experience rather than through crowd size alone.

Social and cultural impacts are explored in a study about a recreational gymnastics event held every year in Cáceres (Spain) (Luis-del Campo et al.). Set within a UNESCO World Heritage city, the study illustrates how a small-scale sporting event can strengthen local identity, enhance a city's visibility, and create opportunities for cultural and social exchange. These benefits can be achieved without many of the disruptions commonly associated with larger events. The event GIMNASTRADA's emphasis on inclusivity and its reliance on existing facilities demonstrate how community participation and cultural sensitivity can contribute to longer-term social sustainability.

The Research Topic also considers emerging forms of sport participation and event consumption such as eSports events. Cerqueira et al. applies the Stimulus–Organism–Response framework to the growing field of eSports revealing that venue atmosphere, facility design, and accessibility shape attendees' emotional responses, which in turn influence intentions to return and recommend the event to others. The article also demonstrates that many of the factors influencing experiences at traditional sporting events such as physical venues, digital expectations, and participant engagement are equally relevant in the eSports context, where physical settings and digital elements increasingly overlap.

In their contribution, Principe et al., have addressed questions of governance and innovation applying the Transformative Blockchain Technological Approaches to sports events*.* Rather than focusing on a specific event, this conceptual contribution develops a framework linking blockchain technology, decentralised autonomous organisations, dynamic capabilities, collaborative governance, and alternative governance models. The article encourages both researchers and practitioners to rethink issues such as transparency, stakeholder involvement, ticketing systems, accountability, and decision-making processes. At the same time, it acknowledges the practical and regulatory challenges that accompany the adoption of decentralised technologies, particularly within smaller event settings.

Environmental sustainability emerges as another important theme in the impact assessment of sport events. In his paper, Perechuda analysed the main challenges and future directions of winter sports events identifying a clear disconnect between broad sustainability ambitions and the realities faced by organisers of grassroots winter sport events. Limited budgets, infrastructure constraints, staffing shortages, unpredictable weather conditions, and snow management challenges for example, complicate sustainability efforts. The study argues that effective sustainability strategies must be grounded in organisational realities and adapted to the specific resources, capacities, and environmental contexts of individual events.

The analysis of community perspectives and destination development was the main topic of the study conducted Post-COVID-19 by Song and Jeong. The purpose was to examine residents' attitudes toward marine sports tourism in the post-COVID-19 era, focusing on the mediating effect of tourism acceptance and the moderating effect of place identity. The study analysed community perspectives and destination development in South Korea and found that residents' knowledge and perceptions of tourism significantly influence their attitudes towards future tourism development. These relationships are further shaped by levels of tourism acceptance and the strength of local place identity. The findings are particularly relevant in the post-pandemic context, where communities continue to balance economic recovery, public well-being, local identity, and sustainable tourism objectives.

The research by Lin et al. provides a final viewpoint on sporting events, highlighting the function of events such as University Marathons as organized health interventions connecting event management with broader educational and public health goals. The article challenges the notion of university marathons as merely competitive events, instead presenting them as platforms for health promotion, community building, and value creation. The authors demonstrate how organisational processes, event culture, activities, and support services contribute to participant engagement, loyalty, and a stronger sense of campus belonging.

The papers in this Research Topic collectively emphasize the idea that medium and small scale sporting events deserve attention in their own right. Their significance lies not in how closely they resemble mega-events, but in their capacity to respond to local contexts and engage local stakeholders. Across the collection, the articles converge on a common insight: the most meaningful impacts are generated through strong connections between events and the communities in which they are embedded. Economic, social, and cultural benefits are therefore best understood as products of thoughtful planning, effective governance, stakeholder collaboration, and positive participant experiences rather than simple descriptive measures and data.

Future research would benefit from more comparative, longitudinal, and mixed-method approaches capable of capturing both immediate outcomes and longer-term legacies. Practitioners and policymakers should consider that, although medium and small scale events require careful planning and management, they offer flexible and adaptable pathways for promoting community development, sustainable sport participation, and active lifestyles. When aligned with local needs, capacities, and aspirations, these events can become powerful tools for creating lasting value.

